# Activation of Molecular Oxygen and Selective Oxidation with Metal Complexes

**DOI:** 10.1021/acs.accounts.4c00731

**Published:** 2025-02-21

**Authors:** Chao Wang, Jianliang Xiao

**Affiliations:** † School of Chemistry and Chemical Engineering, Key Laboratory of Applied Surface and Colloid Chemistry, Ministry of Education, Shaanxi Normal University, Xi’an 710119, China; ‡ Department of Chemistry, University of Liverpool, Liverpool L69 7ZD, United Kingdom

## Abstract

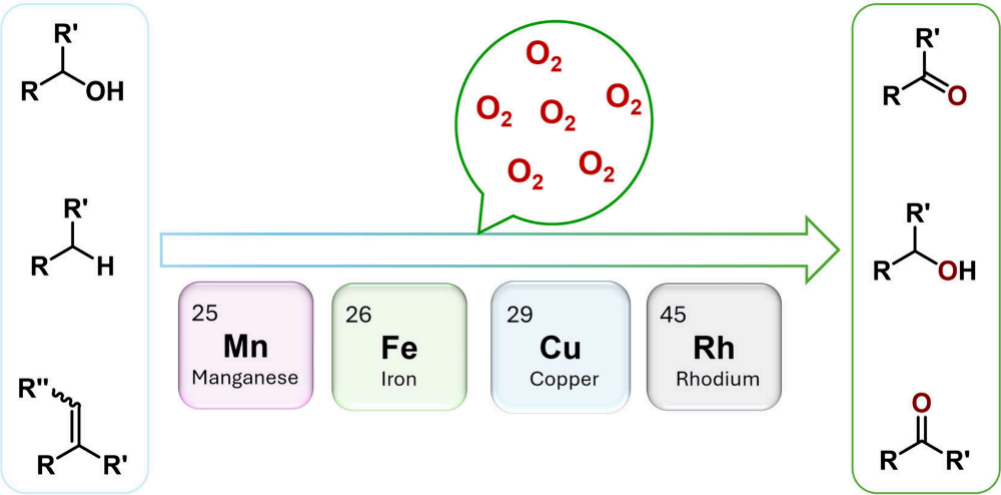

Selective oxidation with molecular oxygen is one of the most appealing approaches to functionalization of organic molecules and, yet at the same time, one of the most challenging reactions facing organic synthesis due to poor selectivity control. Molecular oxygen is a green and inexpensive oxidant, producing water as the only byproduct in oxidation. Not surprisingly, it has been used in the manufacturing of many commodity chemicals in the industry. It is also nature’s choice of oxidant and drives a variety of oxidation reactions critical to life and various other biologic processes. While the past decades have witnessed great progress in understanding, both structurally and mechanistically, how nature exploits metalloenzymes, i.e., monooxygenases and dioxygenases, to tackle some of the most challenging oxidation reactions, e.g., methane oxidation to methanol, there are only a small number of well-defined, man-made metal complexes that have been reported to enable selective oxidation with molecular oxygen of compounds more relevant to fine chemical and pharmaceutical synthesis.

In the past 10 years or so, our laboratories have developed several transition metal complexes and shown that they are capable of catalyzing selective oxidation under 1 atm of O_2_. Thus, we have shown that an Fe(II)-bisimidazolidinyl-pyridine complex catalyzes selective oxygenation of C–H bonds in ethers with concomitant release of hydrogen gas instead of water, and when the iron center is replaced with Fe(III), selective oxidative cleavage of C=C bonds of olefins becomes feasible. To address the low activity of the iron complex in oxidizing less active olefins, we have developed a Mn(II)-bipyridine complex, which catalyzes oxidative cleavage of C=C bonds in aliphatic olefins, C–C bonds in diols, and carboxyl units in carboxylic acids under visible light irradiation. Light is necessary in the oxidation to cleave an off-cycle, inactive manganese dimer into a catalytically active Mn=O oxo species. Furthermore, we have found that a binuclear salicylate-bridged Cu(II) complex enables the C–H oxidation of tetrahydroisoquinolines as well as C=C bond cleavage, and when a catalytic vitamin B1 analogue is brought in, oxygenation of tetrahydroisoquinolines to lactams takes place via carbene catalysis. Still further, we have found that a readily accessible binuclear Rh(II)-terpyridine complex catalyzes the oxidation of alcohols, and being water-soluble, the catalyst can be easily separated and reused multiple times. In addition, we recently unearthed a simple protocol that allows waste polystyrene to be depolymerized to isolable, valuable chemicals. A cheap Brønsted acid acts as the catalyst, activating molecular oxygen to a singlet state through complexation with the polymer under light irradiation, thereby depolymerizing the polymer.

## Key References

- Gonzalez-de-CastroA.; RobertsonC. M.; XiaoJ.Dehydrogenative α-Oxygenation of Ethers with an Iron Catalyst. J. Am. Chem. Soc.2014, 136, 8350–836024835531 10.1021/ja502167h.^1^ An Fe(II) complex was found to catalyze selective oxygenation of C–H bonds in ethers with O_2_, with concomitant release of H_2_.
- HuangZ.; GuanR.; ShanmugamM.; BennettE. L.; RobertsonC. M.; BrookfieldA.; McInnesE. J. L.; XiaoJ.Oxidative Cleavage of Alkenes by O_2_ with a Non-Heme Manganese Catalyst. J. Am. Chem. Soc.2021, 143, 10005–1001334160220 10.1021/jacs.1c05757PMC8297864.^2^ A Mn(II) complex enabled the selective cleavage of C=C bonds of aliphatic olefins to carbonyls with O_2_ under blue light irradiation at room temperature.
- LiuY.; WangC.; XueD.; XiaoM.; LiuJ.; LiC.; XiaoJ.Reactions Catalysed by A Binuclear Copper Complex: Relay Aerobic Oxidation of *N*-Aryl Tetrahydroisoquinolines to Dihydroisoquinolones with A Vitamin B1 Analogue. Chem. Eur. J.2017, 23, 3062–306627880016 10.1002/chem.201604750.^3^ Tetrahydroisoquinolines were oxidized to their corresponding amides through the relay catalysis of a binuclear Cu(II) complex and a vitamin B1 analogue with O_2_ as oxidant.
- WangX.; WangC.; LiuY.; XiaoJ.Acceptorless Dehydrogenation and Aerobic Oxidation of Alcohols with A Reusable Binuclear Rhodium(II) Catalyst in Water. Green Chem.2016, 18, 4605–4610.^4^ A versatile, reusable binuclear Rh(II) complex was found to catalyze both acceptorless dehydrogenation and aerobic oxidation of alcohols in water.

## 1 Introduction

Constituting 21% of the air we breathe and producing water as the only byproduct in oxidation, molecular oxygen, O_2_, is the greenest and most economical oxidant for oxidation reactions. It is used as oxidant in a number of large-scale chemical manufacturing processes; however, its application in fine chemical synthesis, particularly in the pharmaceutical sector, has been limited, where stoichiometric, hazardous oxidants, e.g., KMnO_4_, Na_2_Cr_2_O_7_, OsO_4_, and O_3_, dominated the scene until very recently.^5^ The reluctance to use O_2_ is not only due to safety concerns in sectors lacking experience in using O_2_ but also because of the scarcity of practically useful catalysts, i.e., those that display high selectivity and are easily accessible. The complexity of the mechanisms of O_2_ activation and subsequent oxidation reactions, and hence the difficulty in elucidating the mechanism, add another barrier.^6^

**Scheme 1:**
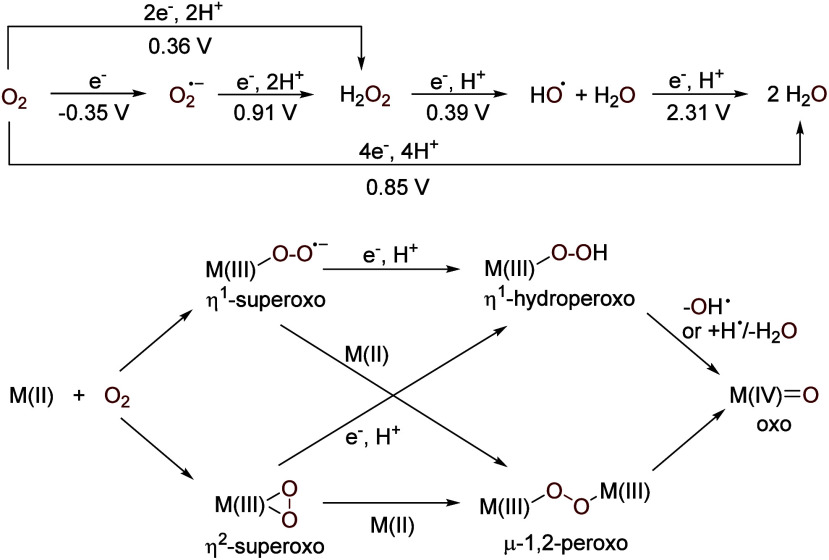
Redox Potential of O_2_ (vs NHE at pH 7) and Schematic Illustration of How It May Be Activated by Metal Complexes

With a high electronegativity in its constituting atom (3.44) and a high standard redox potential (0.85 V, O_2_/H_2_O) (Scheme 1),^7^ the diradical O_2_ is one of the strongest oxidants in nature. Consequently, the reactions of O_2_ with organic molecules are usually highly exothermic, and without precise thermal-catalytic control, over oxidation of the intermediate oxygenated products ensues. However, without an initiator or a catalyst, direct oxidation with O_2_ is kinetically very slow, in general. This is so because of the discrepancy between the ground spin state of O_2_, which is triplet, and that of common organic substrates, which is singlet; that is, the reaction is spin-forbidden.^6a,8^ Looking from a different angle, the unpaired electrons in O_2_ enjoy a high resonance stabilization of ca. 100 kcal/mol (relative to the unpaired electrons in two hydroxyl radicals), thus rendering hydrogen abstraction from a hydrocarbon molecule highly unfavorable.^9^

In contrast, molecular oxygen, being a σ/π donor and acceptor, can readily react with and thereby be activated by transition metal complexes of partly filled d orbitals. As early as 1936 Pauling already noted that “the oxygen molecule undergoes a profound change in electronic structure on combination with hemoglobin”.^10^ It has now been well established that the reaction of O_2_ with metal complexes leads to metal superoxo and peroxo complexes with the dioxygen in various coordination modes, which can further evolve to metal oxo species.^6b−6d,11^Scheme 1 illustrates some commonly seen modes on a mononuclear divalent M(II) center. In most cases, O_2_ activation starts with electron transfer from a low valent metal center, e.g., Mn(II), Fe(II), and Cu(I), to O_2_, giving rise to an η^1^ or sometimes η^2^-superoxo (or peroxo) complex, which can transfer to an η^1^-hydroperoxo species upon hydrogen atom abstraction (HAT) or proton coupled electron transfer (PCET). The single electron reduction of O_2_ has a redox potential that is significantly lower than that of the related two and four electron processes (Scheme 1).^7^ Hence, the formation of metal–superoxo species is thermodynamically unfavorable. Formation of the terminal metal–oxo species necessitates the cleavage of the O–O bond of the superoxo or peroxo intermediates, which usually involves HAT or PCET from a co-reductant or redox-mediator (redox-active cofactor in enzymatic catalysis) and can be facilitated by ligand scaffolds, Lewis acids, and hydrogen bond donors.^12^ However, examples are known of formation of metal–oxo compounds via the intermediacy of peroxide-bridged dimers, through which both oxygen atoms of O_2_ end up in the oxo compounds.^13^ This is highly interesting, as the need for a co-reductant in oxidation becomes redundant, improving process complexity and economy.

Apart from mononuclear metal complexes, multinuclear metal complexes also activate O_2_. The best-known examples are found in nature, e.g., in copper-dependent particulate methane monooxygenase (pMMO) and iron-dependent soluble methane monooxygenase (sMMO), both of which catalyze the remarkable oxidation of methane to methanol under ambient conditions.^14^ Molecular O_2_ can also be converted to reactive oxygen species, such as singlet oxygen, ^1^O_2_, and superoxide, O_2_^•–^, under metal-free conditions.^15^ In the 1990s, one of us was involved in O_2_ activation with metal cluster complexes,^16^ sparking an enduring interest in the activation of O_2_.

Among the various metal–oxygen species, the high-valent metal–oxo is usually the most potent oxidant, capable of oxidizing hydrocarbons of high C–H bond dissociation energy (BDE), such as methane (BDE 105 kcal/mol) to methanol. Such oxo species are generally highly electrophilic in character, activating C–H bonds via HAT, PCET, or hydride transfer (HT) pathways. Metal–superoxo and hydroperoxo species are also oxidizing, but they may display both electrophilic and nucleophilic reactivities in oxidation reactions.^11^ It is these activated oxygen species that provide the opportunities for synthetic chemists to control selective oxidation of organic molecules, as nature does.^17^ Over the past several decades, many metal–oxygen complexes have been synthesized either from O_2_ or other oxidants such as H_2_O_2_, peracids, and PhIO, and their properties have been investigated. A wide range of reviews of their chemistry have been published in the past; more recent ones are seen in the references cited.^18^ However, only a small number of these show viable catalytic activities and selectivities in the oxidation of substrates more relevant to organic and pharmaceutical synthesis with O_2_.

**Scheme 2:**
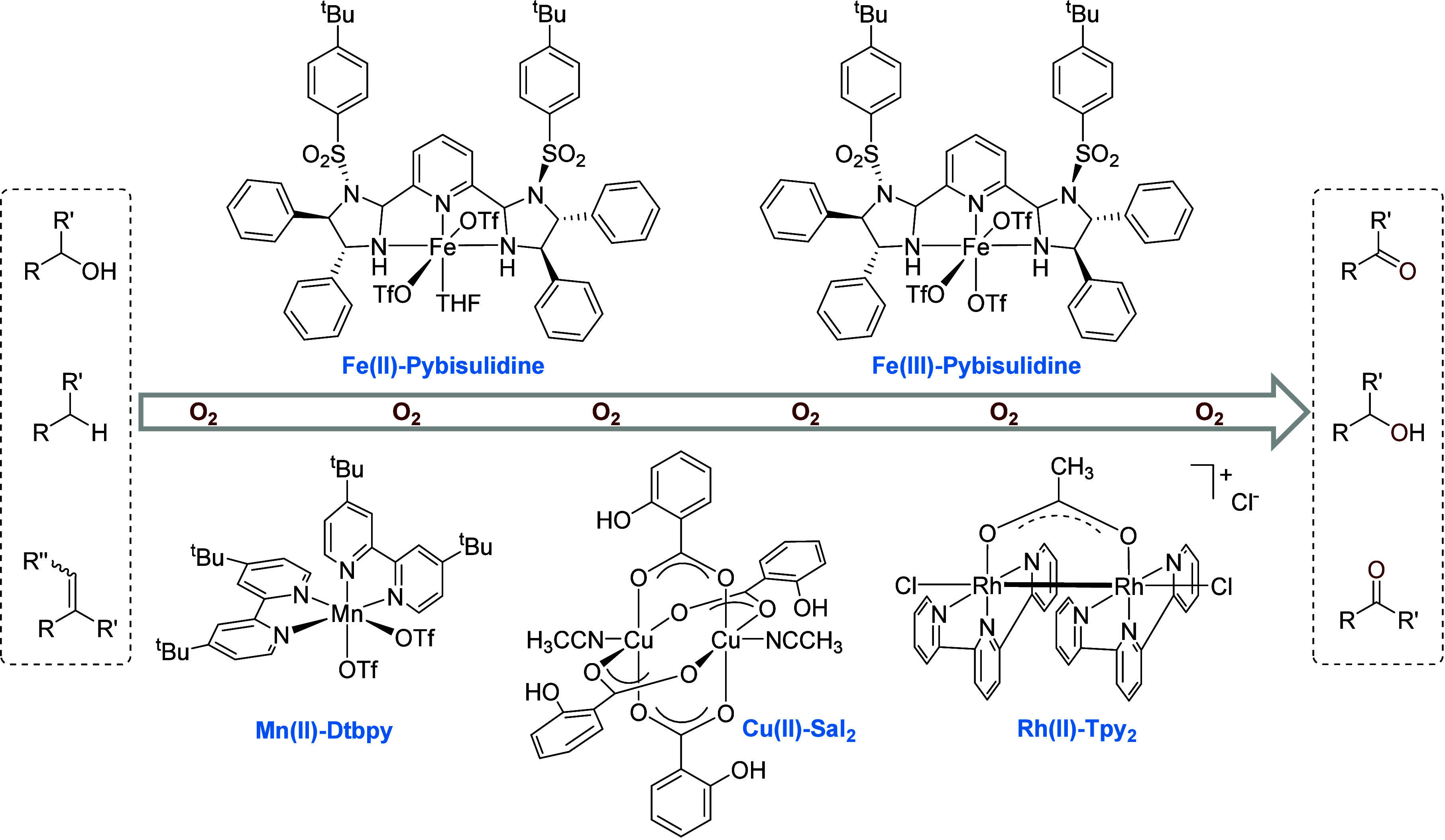
Metal Complexes Shown To Be Active in Catalyzing Selective Oxidation Reactions with O_2_

Driven by the long interest in O_2_ activation, over the past decade our groups have endeavored to achieve selective oxidation, unearthing chemoselective aerobic oxidation reactions with well-defined Fe(II/III), Mn(II), Cu(II), and Rh(II) complexes (Scheme 2). This article attempts to give an account of the work we have carried out. Where possible, references to related recent works from other research groups are given.

## 2 Iron-Catalyzed Oxygenation of Ether C–H and Cleavage of C=C Bonds

Oxygenation of C–H bonds with O_2_ provides a direct approach to accessing alcohols and carbonyls. While a number of metal salts have been shown to catalyze such reactions,^19^ structurally well-defined metal complexes capable of the reaction are limited in number. This has been the case with iron, although the dominance of iron-based oxygenases has inspired generations of chemists to develop biomimetic oxidation catalysts.^6b,17,20^ In the early 2010s, aiming to develop iron catalysts for hydrogenation^21^ we synthesized a series of Fe(II) complexes and found that the complex **Fe(II)-Pybisulidine** (Scheme 2) bearing a sulfonylated pyridine-bisimidazoline ligand (Pybisulidine) showed activities in oxidation of THF to γ-butyrolactone with air.^1,22^ At the time of this finding, there was no synthetic molecular iron complex known for ether oxidation with atmospheric O_2_.

**Scheme 3:**
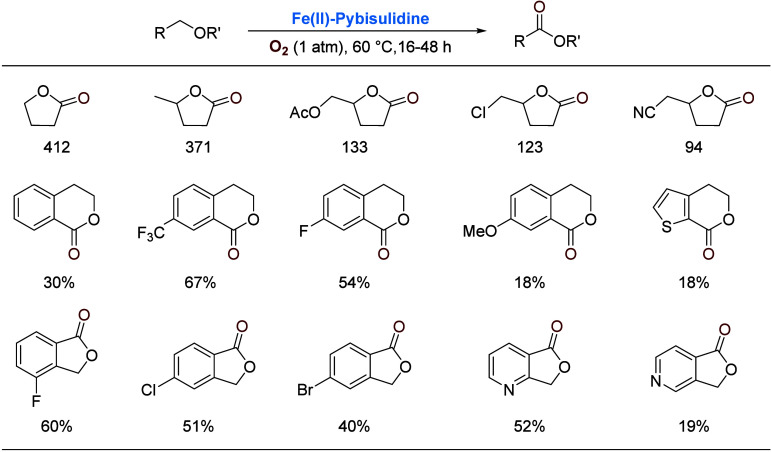
Iron-Catalyzed α-Oxygenation of Ethers Solvent-free reactions. The numbers for γ-butyrolactones refer to TON (48 h) and the percentages to isolated yields (16 h).

Prompted by the scarcity of molecular iron catalysts for such reactions,^23^ we optimized the oxidation and found that **Fe(II)-Pybisulidine** catalyzes oxidation of a ranges of ethers at the α C–H bond under 1 atm of O_2_. Selected examples are listed in Scheme 3. These reactions were carried out without a solvent. While the isolated yields of lactones were low in some cases, the turnover number (TON) was high in general, and more importantly, the catalyst shows good chemoselectivity and functional group tolerance. For both isochromans and phthalans, electron-withdrawing substituents on the aromatic ring induce a higher yield, suggesting that the attacking iron–oxygen species is nucleophilic in reactivity.

**Scheme 4:**
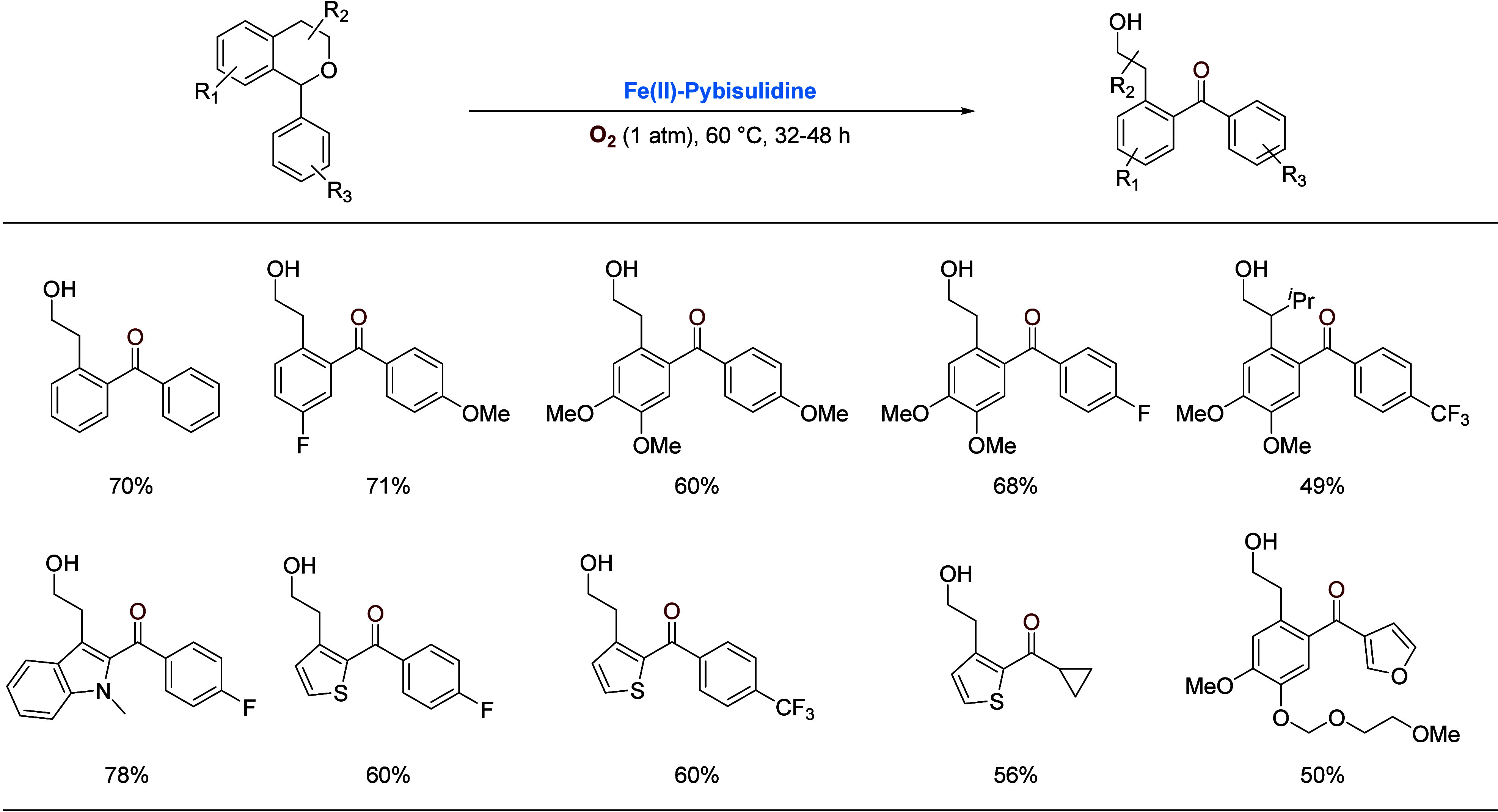
Iron-Catalyzed Oxidation of 1-Arylisochromans and Related Compounds Solvent-free or in C_6_H_6_ under O_2_ (15% v/v in N_2_) (1 atm) at 60 °C with two or three catalyst additions (5.7 × 10^–3^ mmol each), 32–48 h.

When 1-arylisochromans were subjected to the aerobic oxidation, oxidation of the C–H bond as well as cleavage of the endocyclic Csp^3^–O bond was observed, affording valuable 2-(hydroxyethyl)benzophenones (Scheme 4).^24^ Of practical interest is that following three consecutive additions of the catalyst, the product could be obtained in a high isolated yield. Oxidative cleavage of ethereal C–O bonds had only been sporadically studied previously;^23a,23d,25^ laccase enzymes appeared to be the only catalysts known to promote such reactions.^26^

**Scheme 5:**
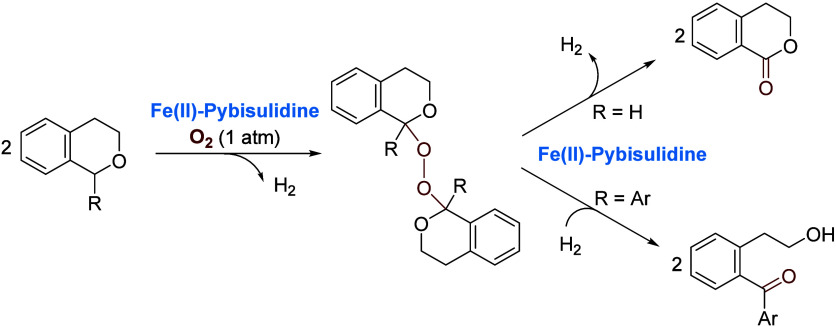
Oxygenation via the Intermediacy of a Peroxide along with H_2_ Release and Reuse

Monitoring the reaction showed that oxidation proceeds via the intermediacy of a peroxide (Scheme 5). The transformation of the peroxide to the ester requires the presence of **Fe(II)-Pybisulidine** but not O_2_. The peroxide originates from one O_2_ molecule, as evidenced by the oxidation with a mixture of ^18^O_2_ and ^16^O_2_, the product of which showed no oxygen crossover. One of the most striking features of the catalysis is the release of a stoichiometric amount of H_2_ in each step of the reaction (Scheme 5). While the mechanism of the oxidation remains unclear, it is likely that O_2_ is activated by the iron catalyst to afford a nucleophilic Fe(III)–superoxo species, which, upon reaction with an ether, gives rise to the peroxide intermediate. The release of H_2_ from the peroxide may proceed via its oxidative addition to **Fe(II)-Pybisulidine** or by a retro [2 + 2 + 2] cycloaddition of the peroxide.^27^ The oxidation of 1-arylisochromans also proceeds via the intermediacy of a peroxide; however, in the absence of an α hydrogen, cleavage of the peroxide bond affords a 2-(hydroxyethyl)benzophenone with the hydroxy proton coming from H_2_ released (Scheme 5).

**Scheme 6:**
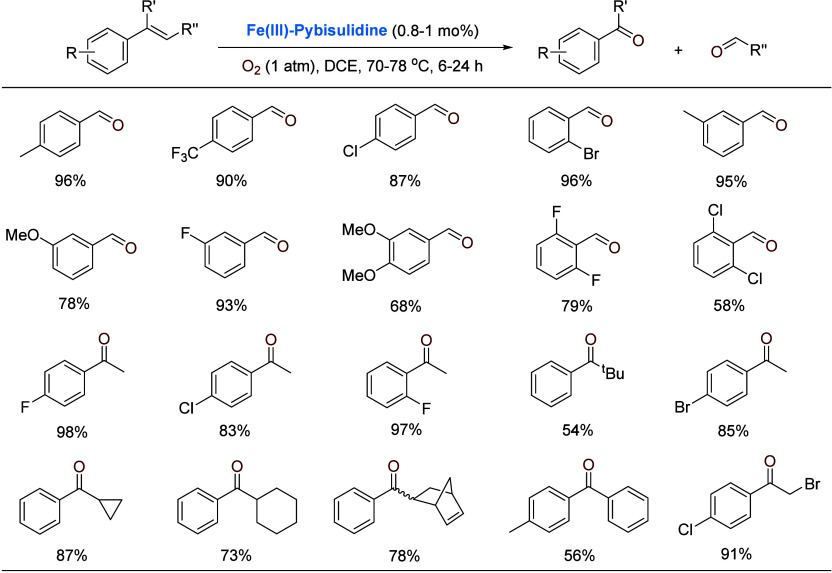
Iron-Catalyzed Oxidative Cleavage of Styrenes 1,2-Dichloroethane (DCE) as solvent.

Oxidative cleavage of alkenes into carbonyls is a widely practiced transformation in synthetic chemistry.^28^ In particular, it has been used in the synthesis of many bioactive compounds,^29^ often via ozonolysis or a stoichiometric metal oxidant that raises safety and waste issues until recently.^30^ While oxygenases are known to cleave C=C double bonds,^31^ synthetic iron catalysts generally necessitate oxidants more active than O_2_.^32^ It was pleasing that the ferric complex **Fe(III)-Pybisulidine** (Scheme 2) was found to be active and chemoselective in aerobic oxidative cleavage of C=C double bonds of styrenes.^33^ Given that Fe(III) is much less likely to activate O_2_ [Fe(III)/Fe(II) 0.77 V, SHE] via electron transfer than Fe(II), somehow counterintuitively, ferrous complex **Fe(II)-Pybisulidine** showed no activity under the conditions tested. Selected examples are shown in Scheme 6.

**Scheme 7:**
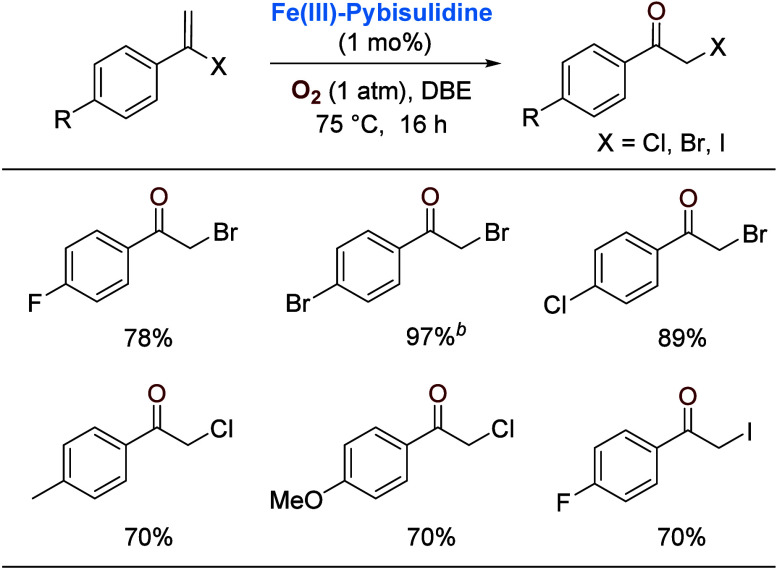
Oxygenation of Vinyl Halides with Concomitant Halogen Migration 1,2-Dibromoethane (DBE) as solvent; DCE as solvent.

Interestingly, when the oxidation was attempted with vinyl halides, migration of the halide to the β carbon was observed, affording α-haloketones (Scheme 7). Earlier, this reaction was found to occur with stoichiometric strong oxidants such as hypohalides.^34^ More recent examples of catalysis have appeared.^35^

**Scheme 8:**
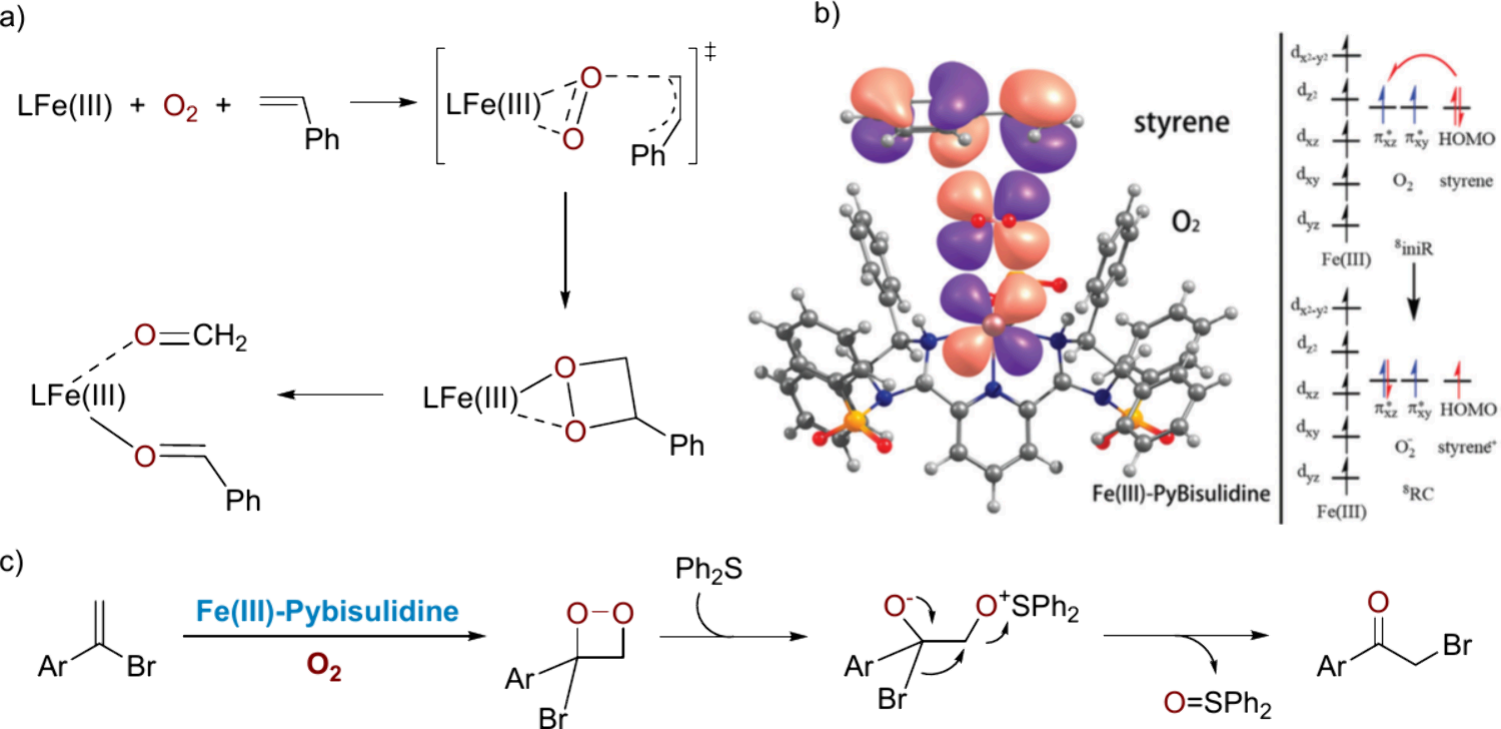
Key Mechanistic Steps in the Oxidative Cleavage of Styrenes (a) DFT-elucidated mode of O_2_ activation and C=C cleavage. (b) Orbital interactions between styrene, O_2_, and iron catalyst (right-hand panel shows orbitals involved in forming ^8^RC from ^8^iniR, with the latter denoting the ground state of the system comprised of catalyst, O_2_, and styrene while the former complex formed upon O_2_ activation; superscript shows spin state) (Reprinted with permission from ref (36), Copyright 2017 Royal Society of Chemistry). (c) Suggested pathway of bromide migration.

The mechanism of the oxidative cleavage has been elucidated by DFT calculations.^36^Scheme 8a,b illustrates the key features of the mechanism. As in our original proposal, the carbonyl products result from C–C cleavage of a dioxetane intermediate. However, its formation proceeds via a transition state in which O_2_ is sandwiched between the ferric center and styrene, rather than involving coordination of both O_2_ and olefin. An intriguing question is how **Fe(III)-Pybisulidine** activates O_2_. The calculation shows that O_2_ is activated via coordination to Fe(III), which renders it more oxidizing and, thus, capable of abstracting an electron from the substrate to form a Fe(III)–superoxo species. Styrene cannot easily transfer an electron to free O_2_ (HOMO of styrene −6.03 eV vs SOMO π* orbital of O_2_ −3.07 eV). However, coordination of O_2_ to the ferric center gives rise to an O_2_ populated LUMO of −8.49 eV, ready to accept an electron from the substrate (Scheme 8b). The electron transfer is further facilitated by the phenyl ring, which stabilizes the resulting carbocation. This mechanism resembles that proposed for the oxidative cleavage of carotenoids by apocarotenoid oxygenase.^37^

The calculations also explain why the ferrous catalyst failed to catalyze the reaction, due to the reduced oxidizing ability of the Fe(II)–O_2_ species, and why aliphatic olefins cannot be oxidized, because of mismatched orbital interactions between the coordinated O_2_ and olefin. The formation of the α-haloketones could also be explained by the intermediacy of dioxetane (Scheme 8c). Since diphenyl sulfide is known to react rapidly with dioxetanes, its introduction increased the yield of α-haloketones significantly, lending support to the purported dioxetane intermediate.

## 3 Manganese-Catalyzed Oxygenation of Olefins, Diols, and Carboxylic Acids

**Scheme 9:**
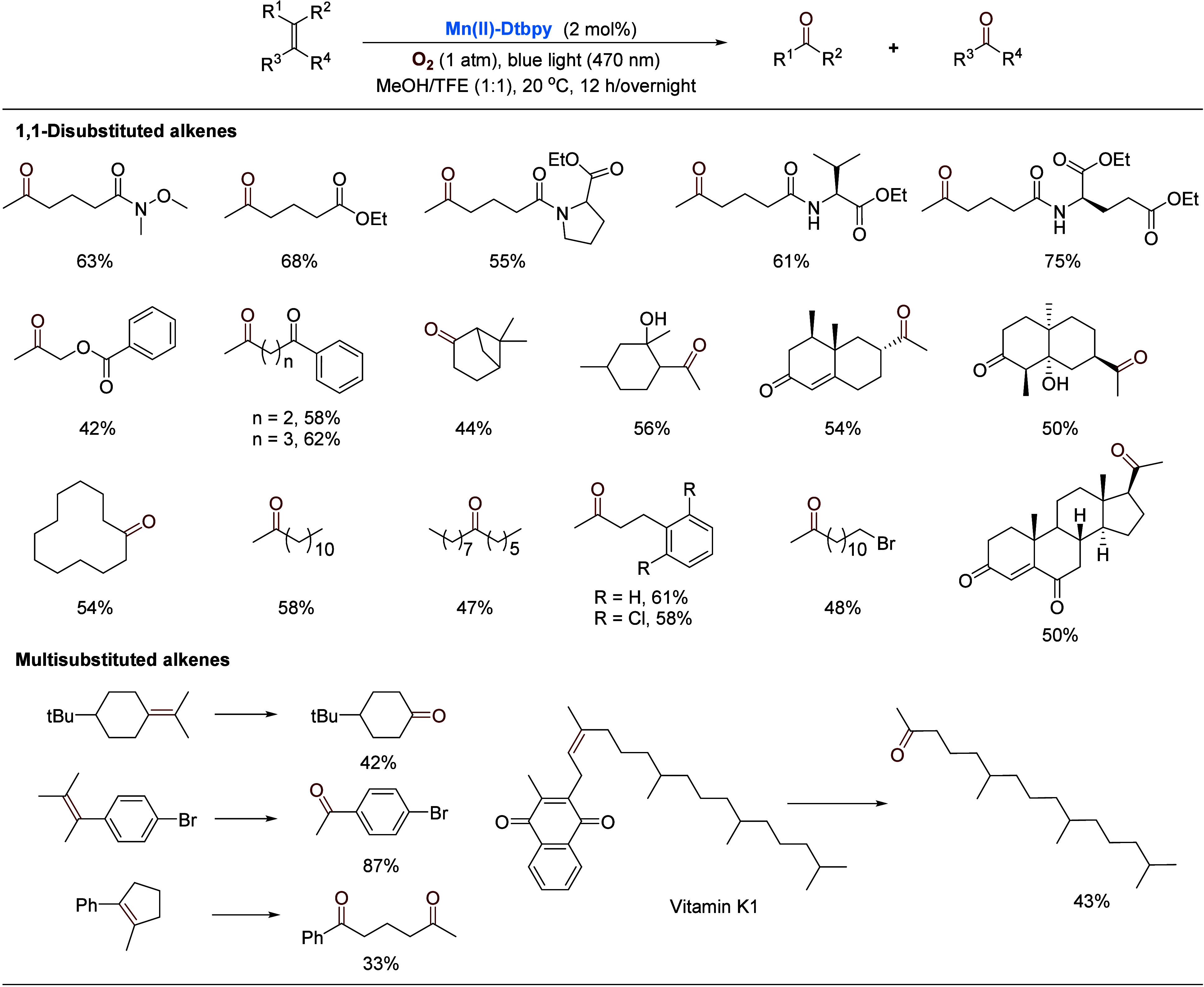
Manganese-Catalyzed Oxidative Cleavage of Olefins

A shortcoming of iron catalysts is their inability to catalyze the oxidative cleavage of more challenging aliphatic olefins. This could partly stem from the inability of aliphatic olefins to transfer an electron to O_2_ (cf. the oxidation potentials of aliphatic olefins and styrenes).^38^ Bearing in mind the amphoteric properties of a photoexcited catalyst being both more oxidizing and more reducing than its ground state, we envisaged a light-driven O_2_ activation strategy for such alkenes.^30e−30h,30j,30l−30n,39^ Upon screening various conditions, we found that the manganese complex **Mn(II)-Dtbpy** (Scheme 2) enables the aerobic oxidative cleavage of aliphatic olefins under blue light irradiation (470 nm, 9 W) in a mixed solvent of methanol and trifluoroethane (TFE) at room temperature.^2^ This was particularly encouraging, considering that manganese is an abundant, biorelevant metal. Further experiments showed that light and methanol are also indispensable. Without a ligand and/or light, Mn(OTf)_2_ is less likely to produce an activated oxygen species from O_2_ [Mn(III)/(II) 1.56 V, SHE]. Selected examples are shown in Scheme 9. A variety of nonactivated 1,1-disubstituted aliphatic alkenes were oxidatively cleaved, including natural products and their derivatives. The oxidation could be extended to internal disubstituted, trisubstituted, and tetrasubstituted alkenes, as well as dialkenes. Notably, vitamin K1 could be oxidized, affording a valuable hexahydrofarnesyl acetone.^40^

**Scheme 10:**
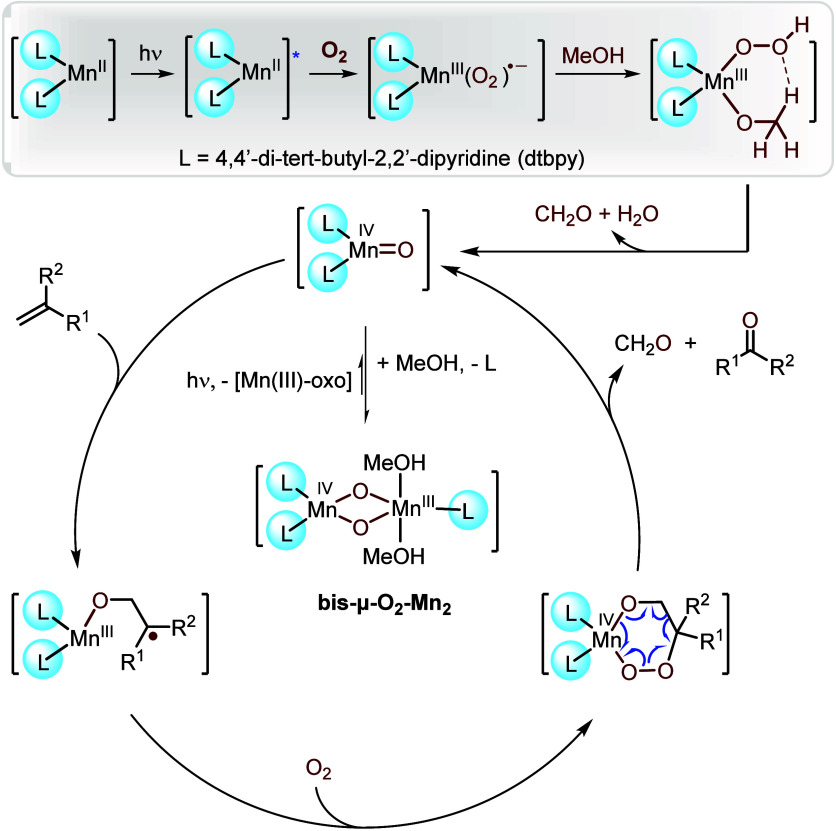
Suggested Mechanism for Manganese-Catalyzed Oxidative Cleavage of Olefins

A putative mechanism for oxidative cleavage is shown in Scheme 10. Under blue light irradiation, **Mn(II)-Dtbpy** is excited and then reacts with O_2_ to form a Mn(III)–superoxo species. The solvent methanol takes up one oxygen atom from the superoxo species, converting it to a Mn(IV)=O oxo species, which starts the catalytic turnover. However, the monomeric oxo can easily form a more stable off-cycle **bis-μ-O**_**2**_**-Mn**_**2**_ complex, thus necessitating continuous irradiation to regenerate the catalytically active oxo species. The mechanism mandates that the two oxygen atoms in the products originate from two different O_2_ molecules. This finds support in an ^16^O_2_–^18^O_2_ tracer experiment, which revealed the formation of all statistically possible products when a mixture of ^16^O_2_–^18^O_2_ was used to oxidize a cyclic olefin.

**Figure 1:**
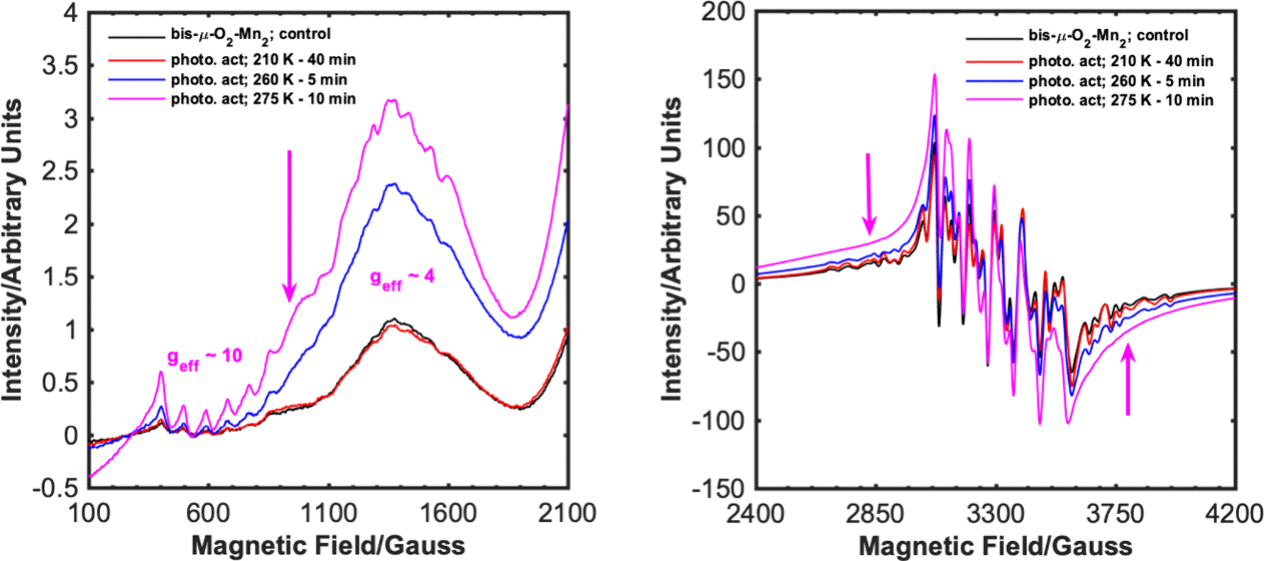
EPR spectra of **bis-μ-O**_**2**_**-Mn**_**2**_ (black trace) dissolved in MeOH (208 K), followed by irradiation with blue light under air for the specified time at the specified temperatures. The left-hand panel shows the zoomed-in *g*_eff_ = 10 and *g*_eff_ = 4–6 regions of the spectra in the right-hand panel.

We were able to isolate the **bis-μ-O**_**2**_**-Mn**_**2**_ complex and determine its structure by X-ray diffraction. Unlike common bis-μ-oxo Mn(III,IV) dimers,^41^ the complex features two asymmetric metal centers in significantly different environments. Notably, the isolated complex shows a clear UV–vis absorption band at 537 nm; the same band was observed when complex **Mn(II)-Dtbpy** was exposed to O_2_ under blue light or reacted with a stronger oxidant, PhIO, indicative of the formation of the same dimer. Furthermore, monitoring a methanol solution of **bis-μ-O**_**2**_**-Mn**_**2**_ with electron paramagnetic resonance (EPR) spectroscopy indicated the generation of high-valent species from the Mn(III/IV) dimer in catalysis (Figure 1). The methanol solution of the dimer gives a complex multiline spectrum, expected for an antiferromagnetically coupled Mn(III/IV) dimer with the *S* = 1/2 ground state. Irradiating the solution with blue light increases the intensity of a signal in the *g*_eff_ = 4 region, with a noticeable enhancement of hyperfine structure (ca. 90 G), along with the development of a pronounced shoulder at ca. 1000 G, while the multiline structure in the *g* = 2 region is lost. The EPR measurements provide clear evidence for the generation of Mn(III) species from the Mn(III/IV) dimer. Although inconclusive, a Mn(IV) monomer (*S* = 3/2) with large zero-field splitting would be expected to give signals in the *g*_eff_ = 4–6 region.^42^

**Mn(II)-Dtbpy** also catalyzes the oxidative cleavage of 1,2-diols via visible light-promoted O_2_ activation.^43^ While the last three decades have witnessed the development of various catalytic methods in aerobic cleavage of 1,2-diols, the environmentally friendly manganese has been much less studied.^44^ Knowing that MeOH can act as the electron and proton donor to fuel O_2_ activation by **Mn(II)-Dtbpy**, leading to its oxidation to formaldehyde and the formation of a high valent Mn(IV)=O species (Scheme 10), we envisaged a substrate-promoted O_2_ activation strategy for the oxidative cleavage of 1,2-diols. First, a diol substrate acts as a two-electron and two-proton donor, reducing one oxygen atom of O_2_ to water, with the other oxygen atom oxidizing Mn(II) to a highly active Mn(IV)–oxo species under light irradiation, which would then react with another diol, leading to its cleavage.^44a,45^ The overall process thus involves the oxidative cleavage of two diol molecules with one O_2_ molecule via two sequential two-electron steps, with water as the only byproduct.

**Scheme 11:**
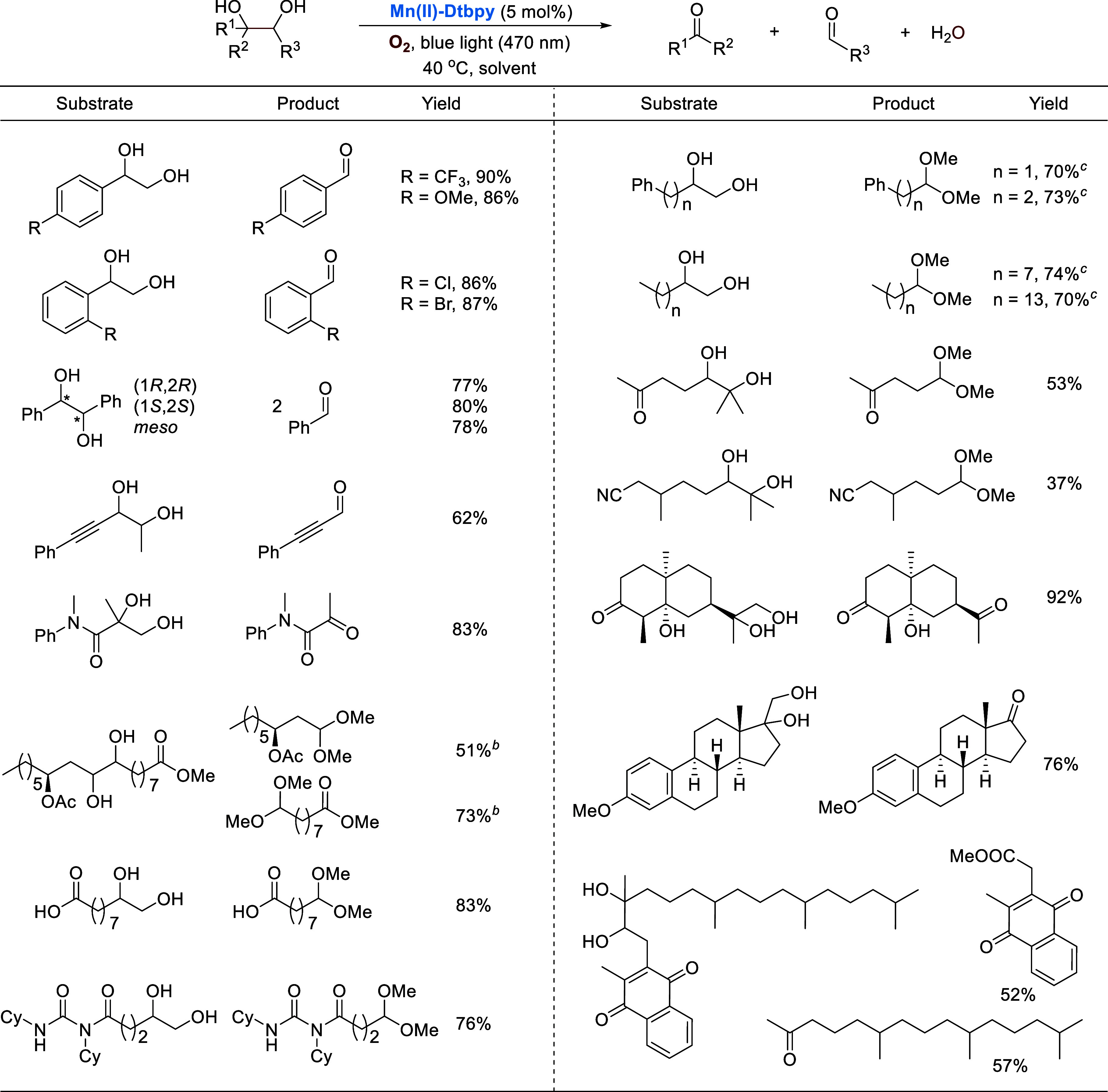
Manganese-Catalyzed Oxidative Cleavage of Diols MeOH or DCE-*t*BuOH (1/3, v/v) as solvent, 13 h. In the case of MeOH, the aldehydes were converted to acetals or ester. Twenty-four hours. Trifluoroethanol added.

The strategy has been shown to work. Selected examples are listed in Scheme 11. A wide variety of diols are tolerated, affording carbonyl products in good yields in general. The oxidation of the more challenging aliphatic diols was carried out in methanol, which presumably promotes the formation of the more active Mn(IV)=O species. The resulting carbonyl products are protected and *in situ* converted to the corresponding acetals. Of note is the tolerance of the catalysis to alkyne and cyclopropane groups, benzylic and C–H bonds, as well as isolated alcohol units, which are prone to oxidation.

**Scheme 12:**
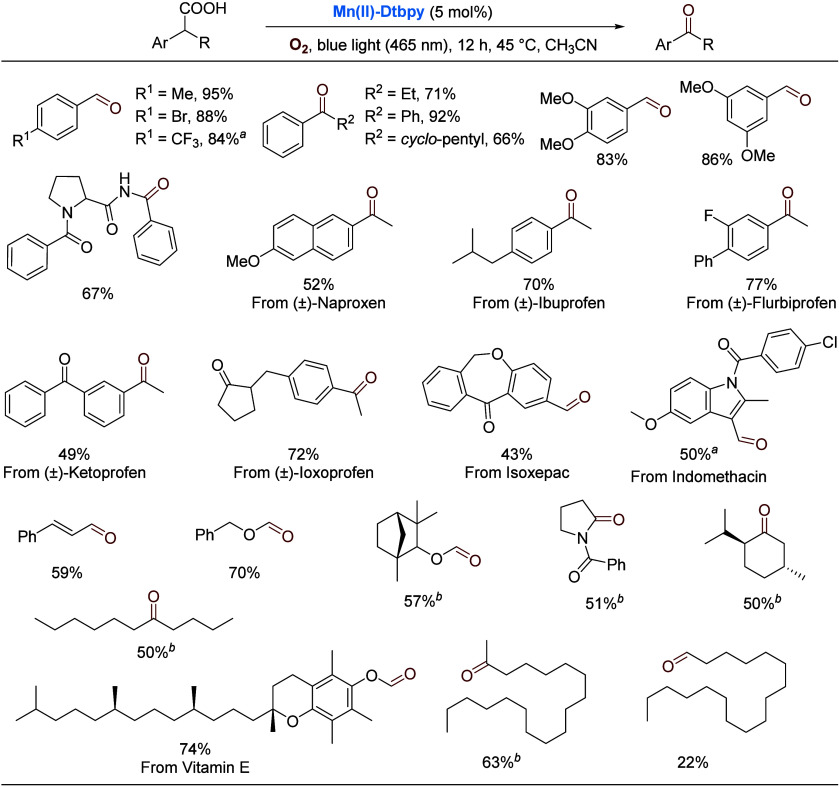
Manganese-Catalyzed Decarboxylative Oxygenation of Carboxylic Acids NaOAc added. DCE–CH_3_CN (1:1, v/v) as solvent, 365 nm light.

**Scheme 13:**
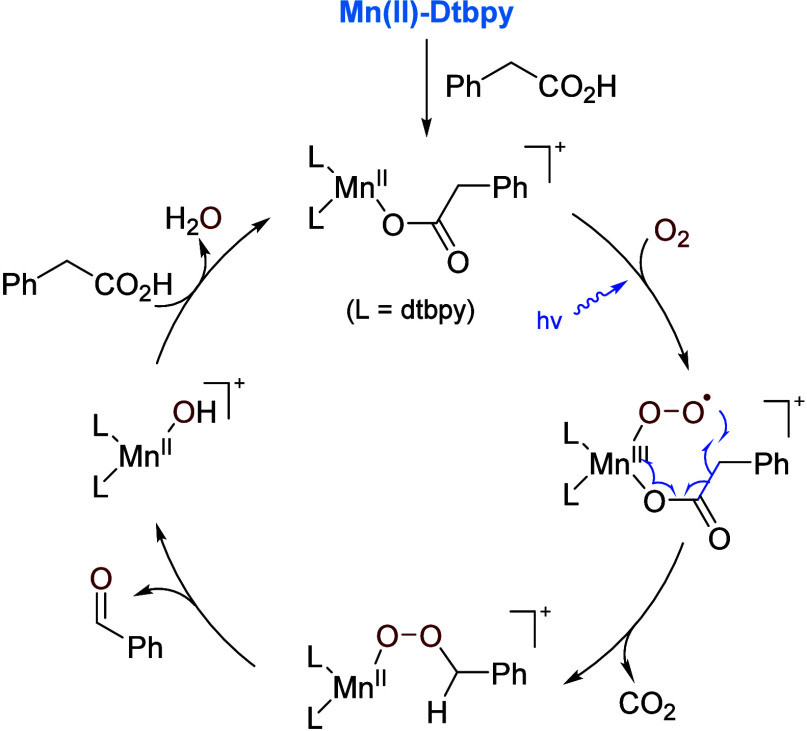
Suggested Mechanism for Decarboxylative Oxygenation of Carboxylic Acids

We have also demonstrated that the manganese complex catalyzes selective decarboxylative aerobic oxygenation of carboxylic acids, one of the most widely available feedstocks in chemical synthesis.^46^ Significant progress has been made in this area in recent years.^47^ With **Mn(II)-Dtbpy** as the catalyst and O_2_ as the oxidant, readily available carboxylic acids can be easily converted to valuable aldehydes, ketones, and amides under blue light irradiation. Selected examples are listed in Scheme 12. Notably, a variety of drug molecules were selectively oxidized by O_2_. For the more challenging aliphatic carboxylic acids, such as fatty acids, UV light (365 nm) was necessary. A purported mechanism is shown in Scheme 13. The key step involves the formation of a Mn(III)–superoxo species under light irradiation, which attacks, driven thermodynamically by the release of CO_2_, the benzylic carbon of the coordinated acid. The resulting Mn(II)–peroxide then decomposes, affording the carbonyl product.

**Scheme 14:**
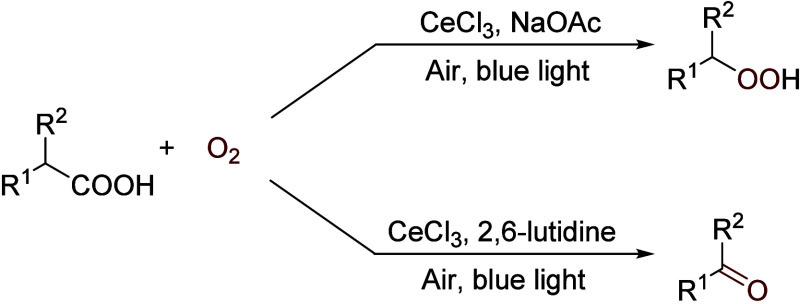
Cerium-Catalyzed Photooxidation of Carboxylic Acids

When using a cerium salt as the catalyst, we have shown that peroxides can be isolated in a synthetically meaningful manner.^48^ Thus, when irradiated under blue light (465 nm, 9 W) in air at room temperature with CeCl_3_ (10 mol %), a range of carboxylic acids were converted to hydroperoxides with good to excellent yields. A base, NaOAc, was needed for the reaction (1 equiv). Remarkably, when the base was replaced with 2,6-lutidine (1 equiv), the oxidation afforded aldehydes/ketones under otherwise the same conditions (Scheme 14). A mechanism involving light-promoted formation of a Ce(IV) superoxide and Ce(III) peroxide species has been suggested.^48^

## 4 Binuclear Copper Catalyzed C–H Oxidation and C=C Bond Cleavage

Enzymes with dicopper active sites are found in important reactions involving O_2_,^14b,49^ and great efforts have been made to design biomimetic binuclear complexes for oxidation.^14b,17a,50^ In 2016, we found that a binuclear paddle-wheel Cu(II) complex, **Cu(II)-Sal**_**2**_ (Scheme 2), could activate O_2_ and catalyze oxidation reactions.^51^ While Cu(II) carboxylate dimer complexes have been studied for their magnetic, electrochemical, and pharmaceutical properties,^52^ their applications in catalysis are rare.

**Scheme 15:**
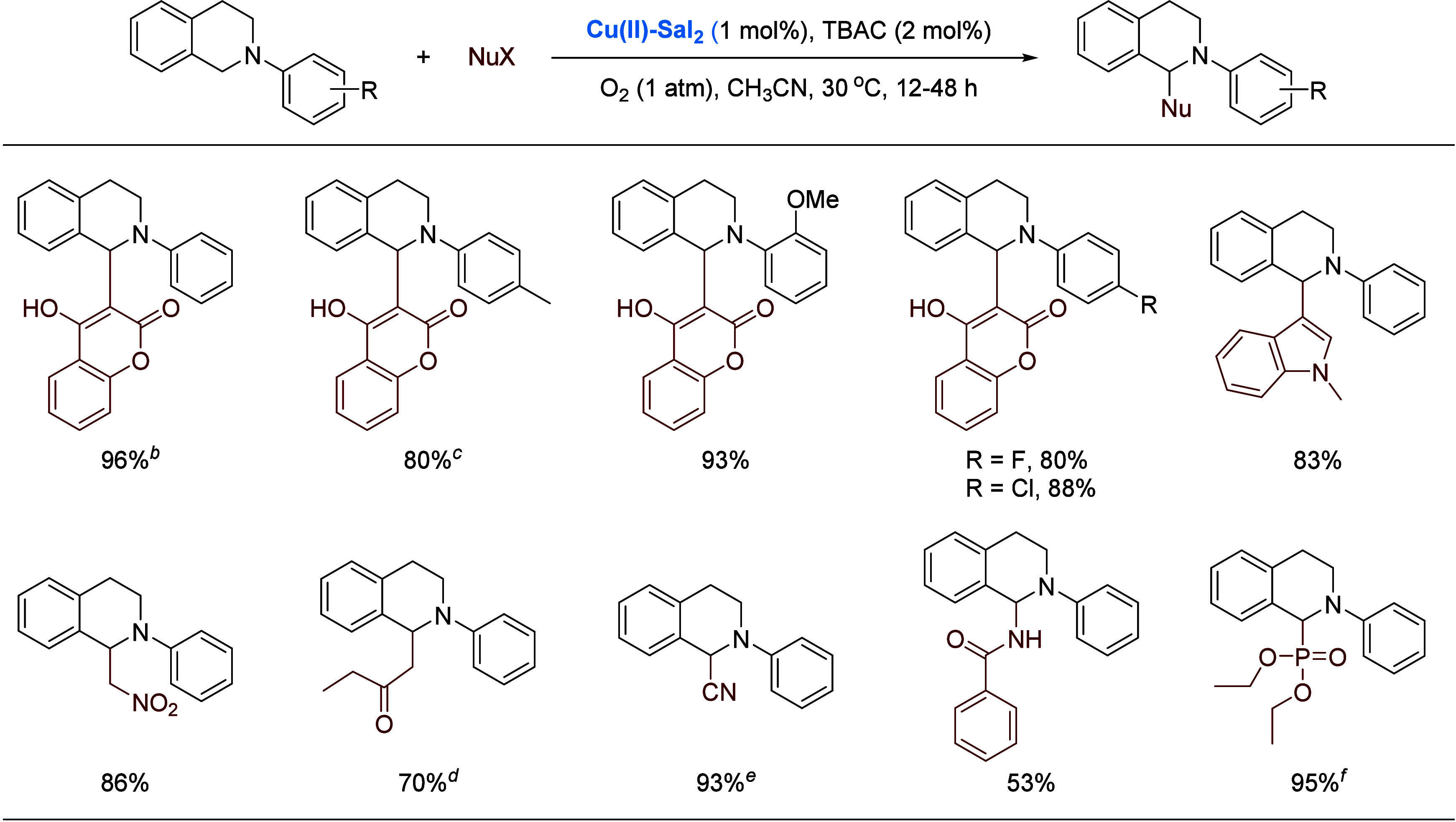
Binuclear Copper-Catalyzed CDC Reaction of Tetrahydroisoquinolines TBAC = *n*-tetrabutyl ammonium chloride. One hour. Four hours. With 10 mol % proline as cocatalyst. Trimethylsilyl cyanide used as prenucleophile. Forty-eight hours.

The cross dehydrogenative coupling (CDC) reaction is a powerful tool for direct functionalization of C–H bonds.^53^ Metal salts such as Cu_2_Cl_2_ and FeCl_3_ as catalysts along with the oxidant *tert*-butylhydroperoxide (TBHP) are often used to effect the reaction. The use of greener oxygen and structurally well-defined catalysts would be desirable. We found that **Cu(II)-Sal**_**2**_ catalyzes the CDC reaction of tetrahydroisoquinolines with various nucleophiles under O_2_. Selected examples are seen in Scheme 15. Note that the CDC is considerably accelerated by a catalytic amount of *n*-tetrabutyl ammonium chloride (TBAC).

Mechanistic studies were conducted to shed light on the CDC. Stoichiometric reactions showed that only in the presence of O_2_ could **Cu(II)-Sal**_**2**_ oxidize a tetrahydroisoquinoline into an iminium intermediate with copper remaining as Cu(II). These observations differ from the simple copper salt-catalyzed CDC reactions, in which the Cu(II) salt could oxidize the amine substrate under an inert atmosphere.^54^ Apparently the salicylate-bridged **Cu(II)-Sal**_**2**_ cannot undergo a two-electron process with the amine without O_2_. Various lines of evidence point to the role of chloride anion in promoting the reduction of Cu(II) to Cu(I).

**Scheme 16:**
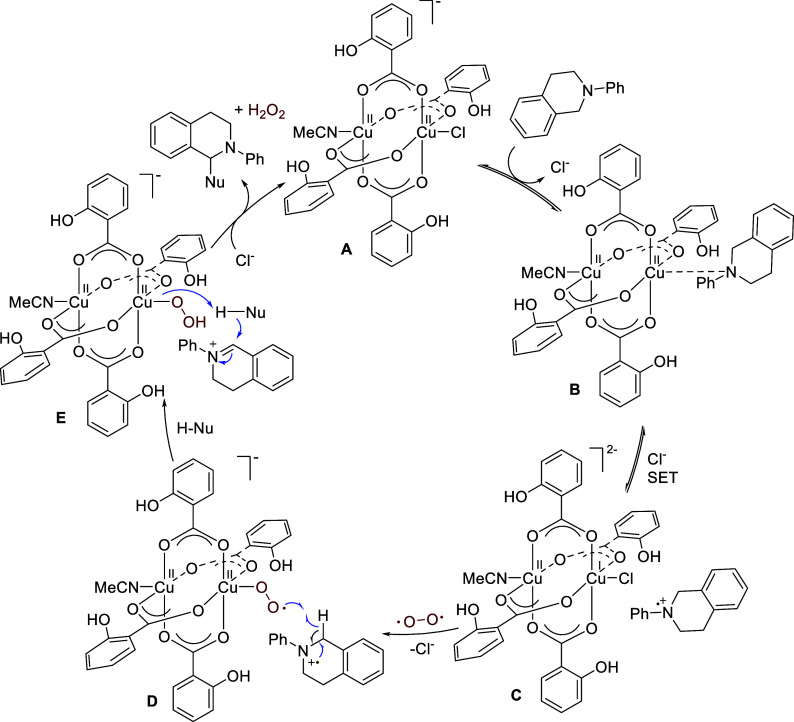
Proposed Mechanism for Copper-Catalyzed CDC Reaction

A plausible mechanism for the binuclear copper-catalyzed CDC is shown in Scheme 16. Coordination of the amine substrate to **A**, which is likely to be in equilibrium with **Cu(II)-Sal**_**2**_, followed by a SET process could form a Cu(II)–Cu(I) intermediate **C** and a radical cation intermediate. DFT calculations suggest that the SET is thermodynamically feasible and kinetically facile.^55^ The SET is reversible, and the presence of the chloride anion presumably shifts the equilibrium to favor **C**. The reversibility of the SET is indicated by the amine substrate remaining intact in the absence of O_2_. The Cu(II)–Cu(I) intermediate then reacts with O_2_ to give the Cu(II)–Cu(II) superoxo radical **D**, which abstracts a hydrogen atom from the amine radical cation to produce intermediate **E**. Subsequently, deprotonation of the nucleophile H-Nu followed by nucleophilic addition to the iminium ion gives the coupling product and one molecule of H_2_O_2_.

**Scheme 17:**
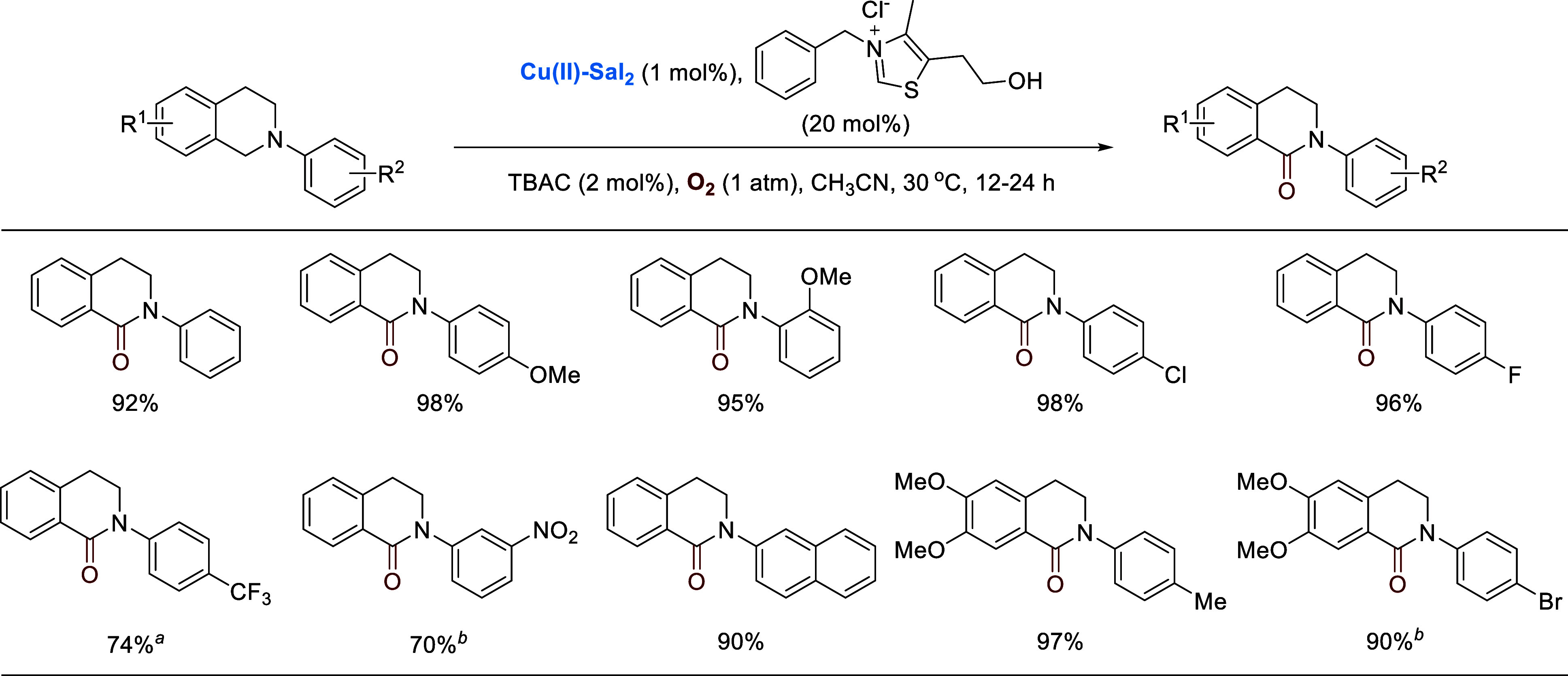
Copper and Vitamin B1 Analogue Co-catalyzed Oxidation of Tetrahydroisoquinolines **Cu(II)-Sal**_**2**_ (10 mol %), 20 mol % TBAC, 60 °C, 96 h. **Cu(II)-Sal**_**2**_ (5 mol %), 10 mol % TBAC, 60 °C.

The complex also catalyzes the oxidation of amines into amides. It was found that in the absence of a nucleophile, *N*-aryltetrahydroisoquinolines could be oxidized to lactams under the catalysis of **Cu(II)-Sal**_**2**_ and a thiazolium salt, a vitamin B1 analogue.^3^Scheme 17 shows selected examples.

**Scheme 18:**
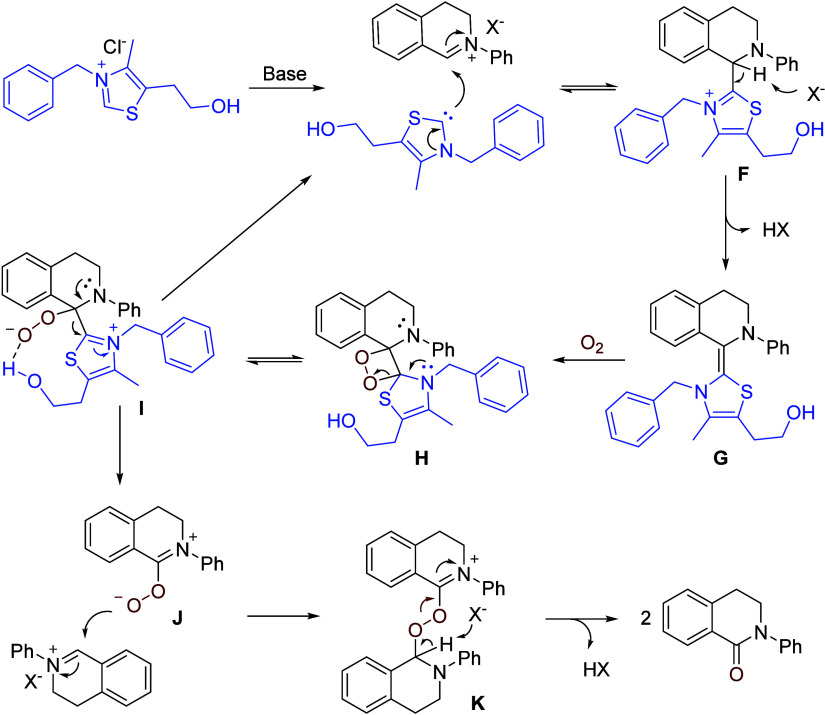
Proposed Mechanism for the Vitamin B1 Analogue-Catalyzed Oxygenation

Mechanistic studies revealed a relay catalytic process. The copper catalyst first converts the amine to an iminium intermediate (Scheme 16), which is then oxygenated by the catalysis of the VB1 analogue. Under the reaction conditions, the thiazolium salt is converted to a carbene, which catalyzes the oxidation of iminium, affording the lactam. The proposed mechanism is shown in Scheme 18. The thiazolium ion is deprotonated to a carbene, which adds to the iminium cation resulting from the oxidation of tetrahydroisoquinoline, generating a Breslow-type intermediate **F**. Deprotonation of **F** leads to an electron-rich ethylenic species **G**, which reacts with O_2_ to form a dioxetane **H** and an equilibrating peroxide **I**. The latter, stabilized by the thiazolium hydroxy group, may readily decompose into the carbene catalyst and the peroxide **J**, which then attacks another iminium cation, forming a peroxo dimer **K**, decomposition of which leads to the lactam product.

The copper catalysis could be extended to the oxidation of *N*,*N*-dimethylanilines.^56^ The reaction leads to methyl oxygenation and oxidative *N*-demethylation. In a separate study, we also showed that **Cu(II)-Sal**_**2**_ catalyzes the aerobic oxidative cleavage of alkenes.^57^ While this reaction had been well documented,^28^ copper catalysis was underdeveloped. It was found that **Cu(II)-Sal**_**2**_ enabled the selective aerobic cleavage of styrene-type olefins, affording ketone or aldehyde products with moderate to good yields. However, as with **Fe(III)-Pybisulidine**, the copper catalyst is ineffective toward less active aliphatic olefins.

## 5 Binuclear Rhodium Catalyzed Oxidation of Alcohols

**Scheme 19:**
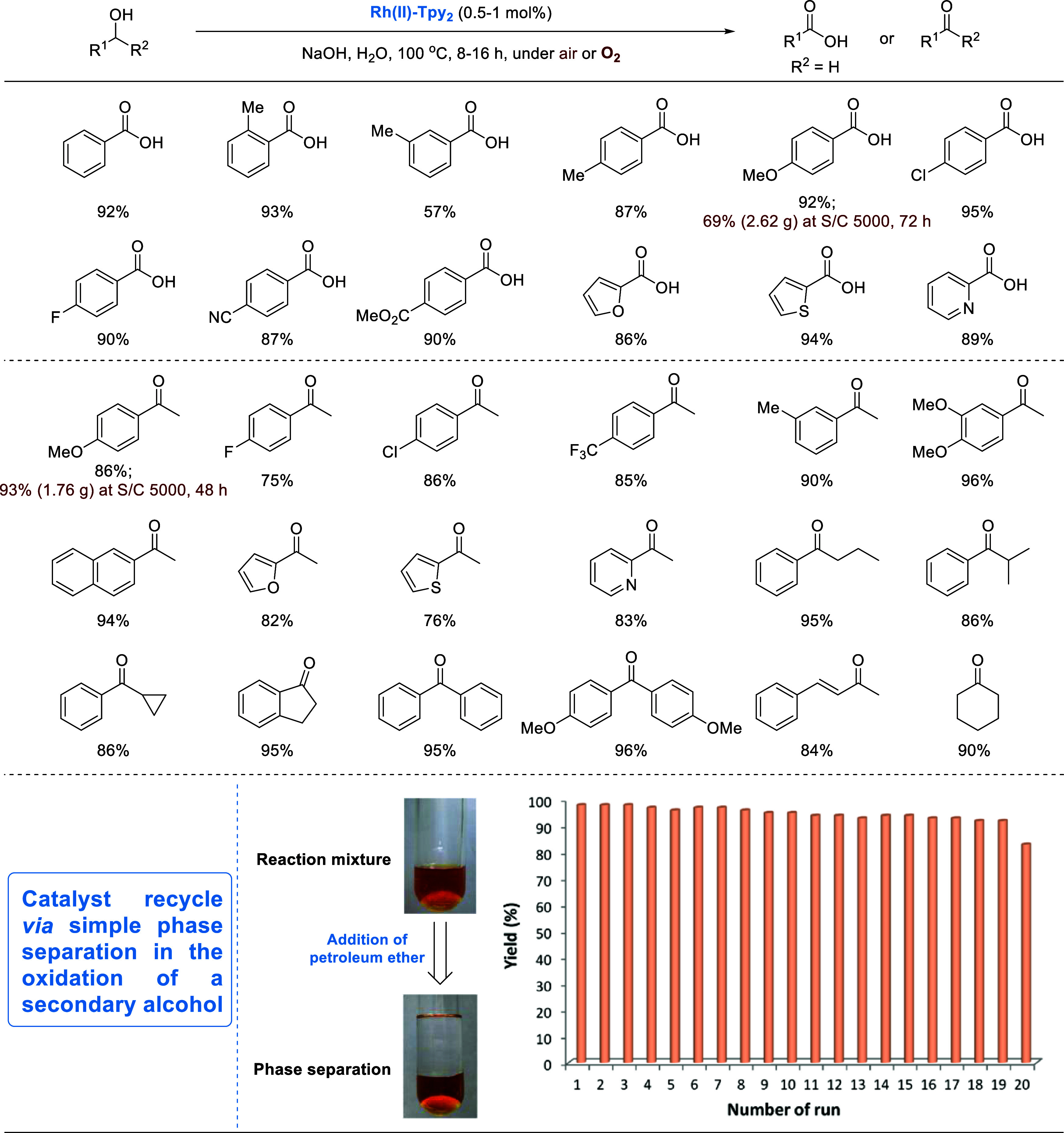
Binuclear Rhodium-Catalyzed Oxidation of Alcohols to Carboxylic Acids and Ketones

Apart from the copper catalyst, we have also found a binuclear rhodium catalyst for the oxidation of alcohols. In 2016, we serendipitously discovered that the readily available binuclear rhodium complex **Rh(II)-Tpy**_**2**_ (Scheme 2) catalyzes the acceptorless dehydrogenative cross coupling of alcohols.^58^**Rh(II)-Tpy**_**2**_ is a water-soluble complex and was later found to catalyze efficient oxidation of alcohols into carboxylic acids or ketones.^4^ The reaction proceeds via dehydrogenation of the alcohol to form a rhodium hydride species, which can be protonated to release H_2_ or oxidized by O_2_ to form water. Thus, the oxidation could take place under either an argon or air atmosphere. Scheme 19 shows examples of the aqueous-phase aerobic oxidation of primary alcohols into carboxylic acids and secondary alcohols into ketones. Being water-soluble, **Rh(II)-Tpy**_**2**_ could be reused multiple times, as demonstrated by the oxidation of 1-(4-methoxyphenyl)ethan-1-ol.

**Scheme 20:**
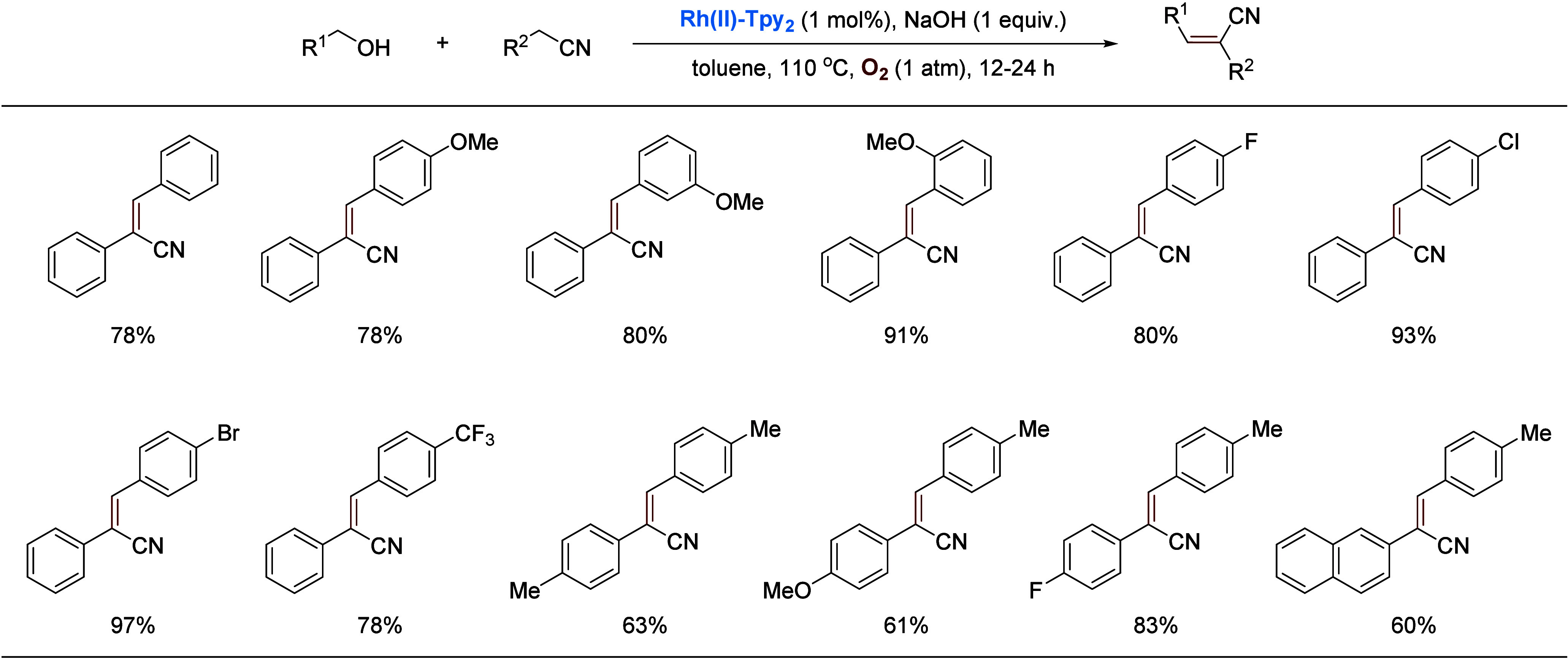
Binuclear Rhodium-Catalyzed Olefination of Nitriles with Alcohols

The Rh(II) catalyst could also be used for the selective alkylation or olefination of alkylnitriles with alcohols, depending on the reaction conditions. Under the catalysis of **Rh(II)-Tpy**_**2**_, an alcohol is dehydrogenated to an aldehyde, which goes on to alkylate a nitrile, affording an unsaturated alkylated nitrile compound. When the reaction is conducted in an inert atmosphere, the unsaturated nitrile will be reduced by the rhodium-hydride generated from the dehydrogenation; however, when run under O_2_ which oxidizes the hydride, the unsaturated nitrile remains intact. Scheme 20 shows examples of the **Rh(II)-Tpy**_**2**_ catalyzed olefination of alkylnitriles with alcohols, affording α,β-unsaturated nitriles.^59^

## 6 Brønsted Acid-Catalyzed Aerobic Oxidation of Polystyrene

**Scheme 21:**
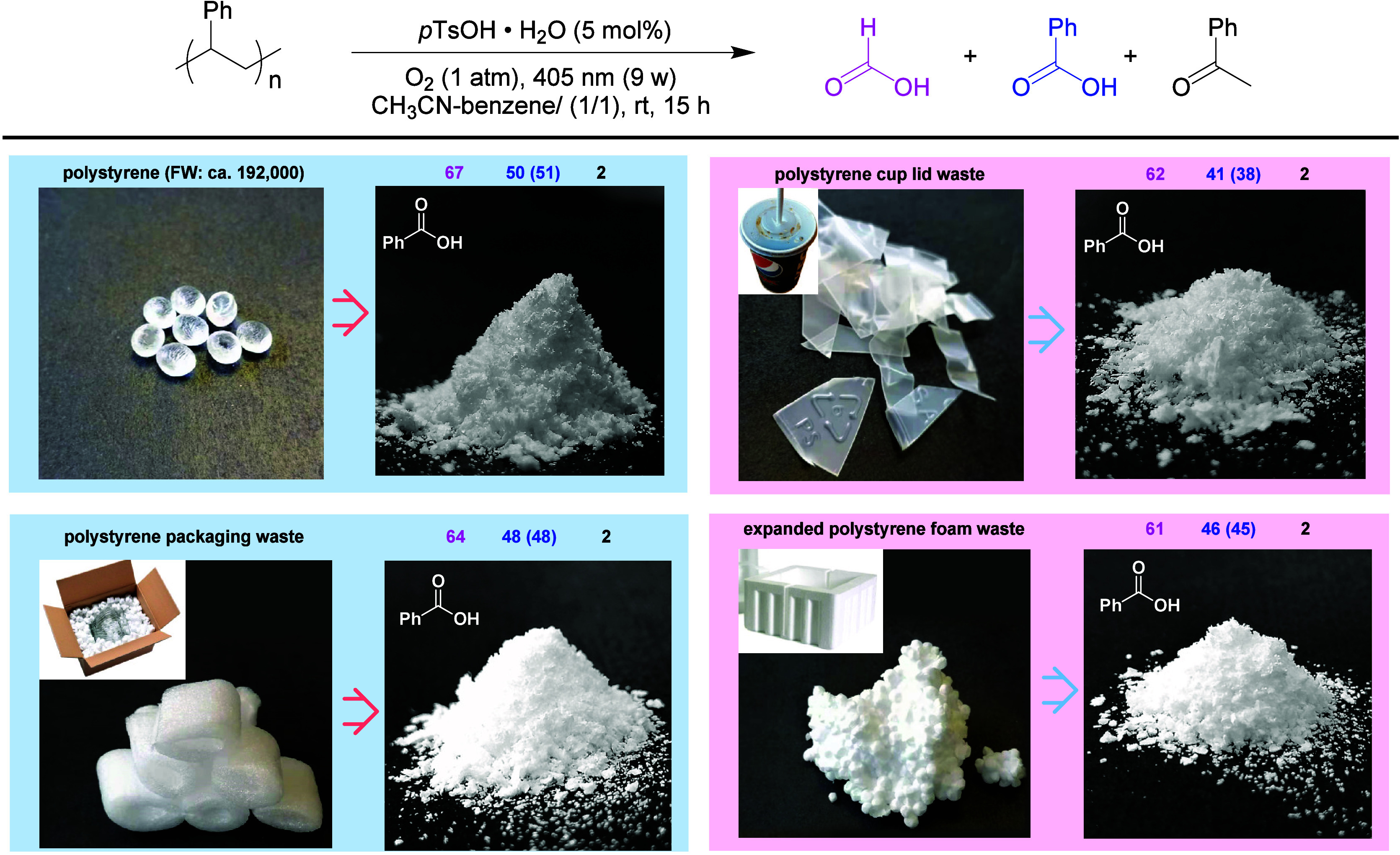
*p*-Toluenesulfonic Acid-Catalyzed Oxidative Depolymerization of PS with O_2_ Yields of products are based on the single repeat unit of PS; NMR yields are given on top of each image identifiable by color; isolated yields in parentheses.

Polystyrene (PS) is one of the most important plastic materials, accounting for about 5% of the global plastic market share in 2021.^60^ While chemical recycling of PS waste has been extensively investigated recently,^61^ in the decades prior to early 2020s, there had been only a few examples of PS degradation via catalytic oxidation.^62^ In continuing our endeavor in oxidation, we found a simple degradation method in 2022, which enables selective oxidative cleavage of PS by O_2_ under acid catalysis and light irradiation (Scheme 21).^63^

In arriving at the approach, we screened a range of potential catalysts, including the metal complexes above. The key observations were that O_2_, a strong catalytic acid and light irradiation (405 nm) are sufficient for the degradation. Neither a metal catalyst nor a photosensitizer was needed. We chose *p*-toluenesulfonic acid (pTsOH·H_2_O), as it is easily available at various scales, environmentally friendly, and easy to handle.^64^

Under the conditions established, PSs of different molecular weights could be oxidatively cleaved to afford formic acid in over 50%, benzoic acid in ca. 35–50%, and acetophenone in 2–5% yields. Remarkably, various types of PS waste could be readily degraded (Scheme 21). It is worth noting that benzoic acid was isolated as a pure white crystalline powder, while formic acid could be converted to isolable formanilides by the addition of an amine. The practical applicability of the protocol was further enhanced using continuous-flow technology.

**Scheme 22:**
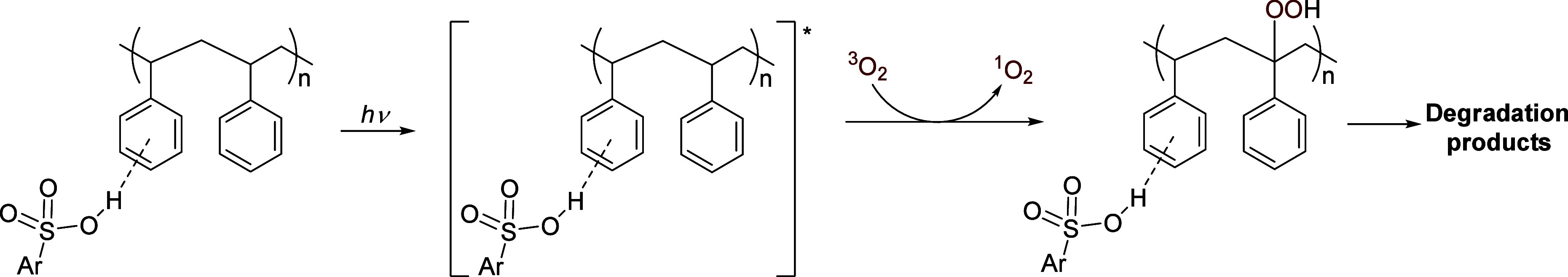
Schematic Illustration of How PS May Be Degraded by O_2_ under Light and Acid Catalysis

Mechanistic studies show that the oxidizing agent in the reaction is ^1^O_2_, derived from the triplet O_2_ used. This is supported by probe reactions with ^1^O_2_ scavengers and *in situ* EPR measurements, including spin trapping. UV–vis measurement of a mixture of PS and *p*TsOH·H_2_O revealed an absorption band at around 408 nm. Further, the isolated byproduct shows a strong absorption at 405 nm. These observations suggest that the acid may interact with PS, e.g., forming a complex with PS via cation−π interactions,^65^ which acts as a photosensitizer to initiate the formation of ^1^O_2_ under the irradiation of light. DFT calculations show that the resulting ^1^O_2_ can easily abstract the hydrogen atom from the tertiary benzylic C–H bond, initiating degradation of the PS chain (Scheme 22).

## 7 Conclusion

Selective oxidation with molecular oxygen is the most appealing pathway to introduce an oxygen-based functionality into a hydrocarbon compound. This Account summarizes the efforts we have made in this fascinating area of research. By complexing iron, copper, manganese, and rhodium salts with tailored or commercially accessible ligands, the resulting molecular complexes have been shown to catalyze a wide range of oxygenation reactions under 1 atm of O_2_. Mechanistically, it is interesting to note that the iron-catalyzed ether oxidation releases H_2_ instead of forming H_2_O, light can be harnessed to reintegrate an off-cycle manganese dimer back into the catalytic cycle, and the binuclearity of the copper complex aids in the oxidation. Additionally, a vitamin B1 analogue can be cascaded with copper to allow for O_2_ activation and amine oxygenation. However, these complexes have come to light mainly by serendipity rather than de novo design. It remains highly challenging to predict what metal and ligand combination would lead to a metal complex that can activate O_2_ and enable chemo- and site-selective oxidation of a substrate. The intricate oxidation reactions in nature are predominately catalyzed by iron-, copper-, and manganese-based enzymes, from which chemists can continuously garner insight and inspiration to design better catalysts for selective oxidation.
